# Supplementary material for: MALT-1 mediates IL-17 neural signaling to regulate C. elegans behavior, immunity and longevity
Source: Nat Commun. 2020 Apr 29;11:2099. doi: 10.1038/s41467-020-15872-y (PMC7190641; doi:10.1038/s41467-020-15872-y)
Supplement: Supplementary file 1 — Supplementary Information [file 41467_2020_15872_MOESM1_ESM.pdf]

MALT1 MEDIATES IL-17 NEURAL SIGNALING TO REGULATE *C. ELEGANS* BEHAVIOR, IMMUNITY AND LONGEVITY

Flynn et al.

Supplementary Information

Supplementary Figures

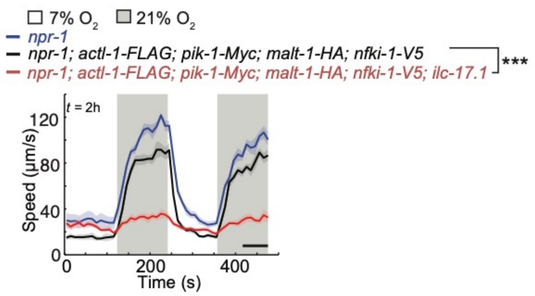

Supplementary Figure 1. Related to Figure 1. Knocking in epitope tags into endogenous *actl-1*, *pik-1*, *malt-1*, and *nfki-1* does not disrupt their function**.**

A strain co-expressing *actl1-1-HA*, *pik-1-Myc*, *malt-1-HA* and *nfki-1-V5* in an *npr-1* background responds to 21% O_2 like *npr-1* controls. Arousal is abolished when these knock-in alleles are crossed into an *ilc-17.1(tm5218)* background. n = 69 animals (*npr-1*), n = 81 animals (*npr-1; actl-1-FLAG; pik-1-Myc; malt-1-HA; nfki-1-V5*), n = 113 animals (*npr-1; actl-1-FLAG; pik-1-Myc; malt-1-HA; nfki-1-V5; ilc-17.1*) ***, P = 1.18e-27, Mann-Whitney *U* test.

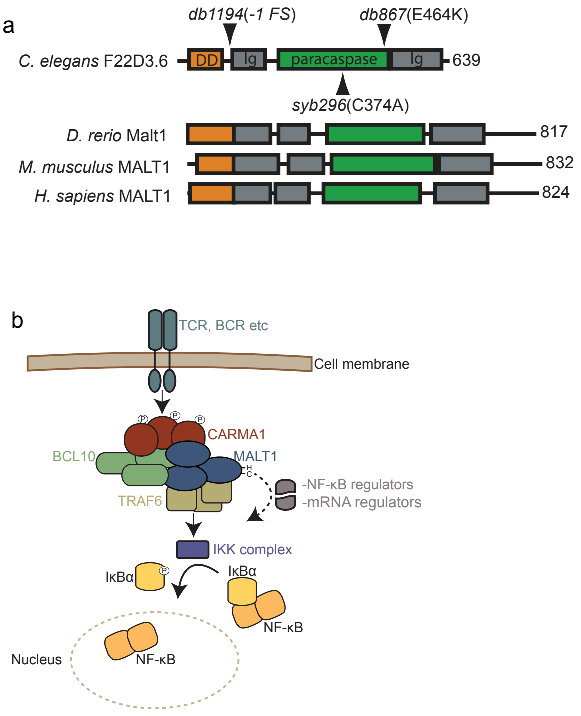

Supplementary Figure 2. Related to Figure 1. MALT1.

a MALT1 paracaspase domain organization. Black arrows indicate the impact of *malt-1* mutations. DD, death domain; Ig, Immunoglobulin-like fold.

b Schematic of MALT1 signaling in mammalian B and T cells. BCR/TCR stimulation induces formation of the CARMA1-BCL10-MALT1 (CBM) signalosome via protein kinase C β (PKCβ)/PKCθ-mediated phosphorylation of CARMA1. MALT1 recruits TRAF6, whose ubiquitin ligase activity leads to the recruitment and activation of the IKK complex. This culminates in IKK-mediated phosphorylation of IκBα, triggering its degradation and releasing NF-κB for nuclear translocation. MALT1 also promotes lymphocyte activation by cleaving negative regulators of NF-κB, and factors that regulate mRNA stability. Blast searches do not find nematode orthologs of BCL10 or CARMA.

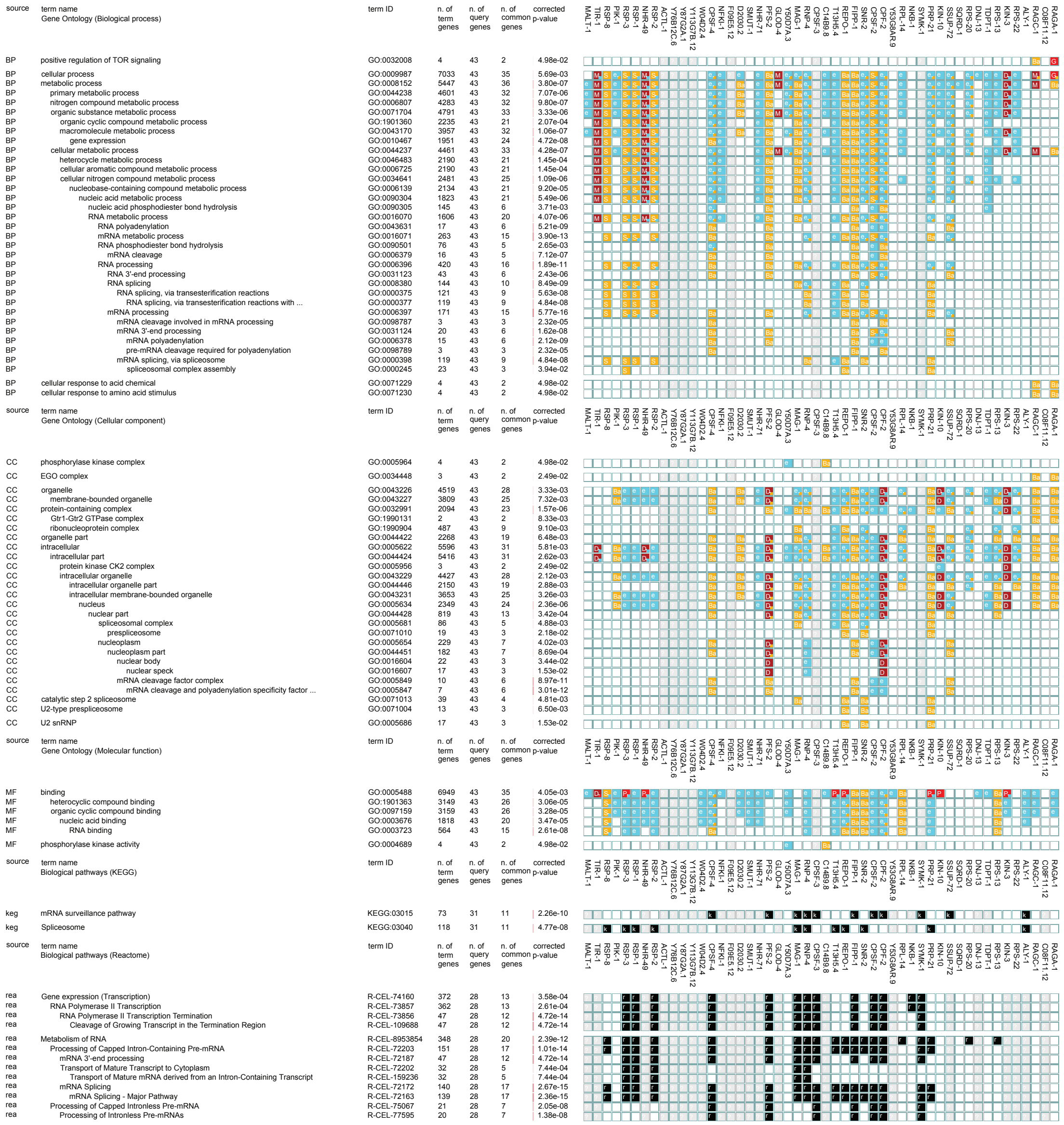

Supplementary Figure 3. Related to Figure 1. The MALT-1-interacting proteome is enriched in factors involved in RNA metabolism.

Gene ontology and biological pathway enrichment analysis of the 50 factors that specifically co-immunoprecipitate with MALT-1 (Supplementary Data 1), performed with g:Profiler[79].

a

Strain	Position	Reference	Alteration	Effect	aa change
AX3324	6948747	G	A	STOP_GAINED	W495*
AX3621	6948842	G	A	NON_SYNONYMOUS_CODING	E464K
AX3713	6948859	G	A	NON_SYNONYMOUS_CODING	C458Y
AX3868	6951060	G	A	NON_SYNONYMOUS_CODING	G63R

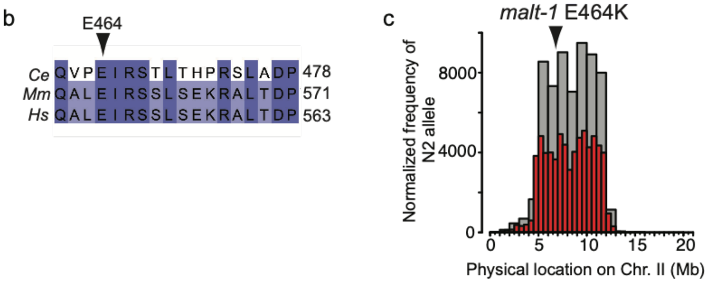

d

Strain	Allele	Position	Reference	Alteration	Effect	aa change
AX5877	*db1194*	6950732	CCACCG-	--ACCTA	FRAME_SHIFT	-1

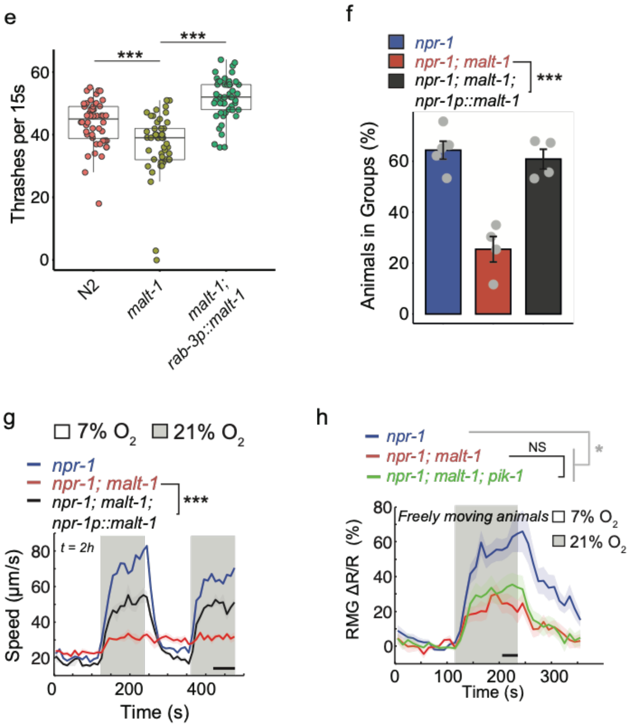

Supplementary Figure 4. Related to Figure 1. MALT-1 promotes escape from 21 O_2.

a *malt-1* mutations isolated in a forward genetic screen for loss of aggregation behavior.

b The MALT-1 E464 residue mutated in AX3621 is conserved from *Ce* to human, and corresponds to E549 in human, which is known to be required for proteolytic activity. *Mm* = *Mus musculus, Hs* = *Homo sapiens*.

c The aggregation defect of AX3621 maps close to *malt-1*. Mapping used CloudMap (see Methods), which measures the relative levels of SNPs derived from two genetic backgrounds in aggregation-defective recombinants. N2 single nucleotide polymorphisms (SNPs) are derived from AX3621; Hawaiian SNPs are

derived from the AX288 (*lon-2 npr-1*) Hawaiian background strain. Aggregation-defective recombinants show an enrichment of N2 Bristol SNPs on chromosome II, flanking the physical location of the *malt-1* mutation at 6.9 Mb.

d The *malt-1(db1194)* allele, generated using CRISPR/Cas9 genome editing.

e *malt-1* mutants exhibit minor defects in thrashing rate. Data are presented as median (centre) and interquartile range (box; the lower and upper bounds of the box represent the 25th and 75th percentiles respectively). Whiskers represent ±1.5x interquartile range. n = 48 animals. ***, P < 0.001, one-way ANOVA with Tukey's post hoc HSD.

f and **g** The aggregation phenotype (**f**) and arousal defect in 21% O_2 (**g**) of *malt-1(db1194)* mutants is rescued by expressing *malt-1* cDNA from the *npr-1* promoter, which drives expression in a broad subset of neurons including URX and RMG[29,47]. (**f**) N = 5 assays (*npr-1)*, N = 4 assays (*npr-1; malt-1*) and (*npr-1; malt-1; npr-1p::malt-1*), ***, P < 0.001, ANOVA with Tukey's post hoc HSD (**g**) n = 44 animals (*npr-1*), n = 84 animals (*npr-1; malt-1*), n = 77 animals (*npr-1; malt-1; npr-1p::malt-1*). ***, P = 5.33e-08, Mann-Whitney *U* test.

h *malt-1* RMG Ca^{2+} transients (reported by YC2.60) in freely moving animals are not further reduced by loss-of-function mutations in *pik-1*. n = 20 animals (*npr-1*), n = 17 animals (*npr-1; malt-1*), n = 17 animals (*npr-1; malt-1; pik-1*). NS, P = 0.3, *, P < 0.05, two-sided Mann-Whitney *U* test.

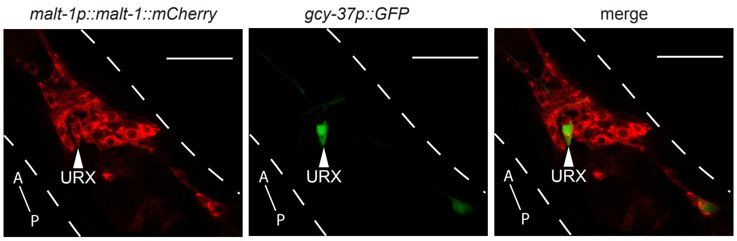

Supplementary Figure 5. Related to Figure 2. MALT-1 is expressed in O_2-sensing neurons. MALT-1::mCherry translational fusion, expressed from its endogenous promoter (4kb), is expressed in URX neurons in the head which are labelled with a *gcy-37p::gfp* reporter. Similar results were obtained in two experiments. Scale bars: 20µm.

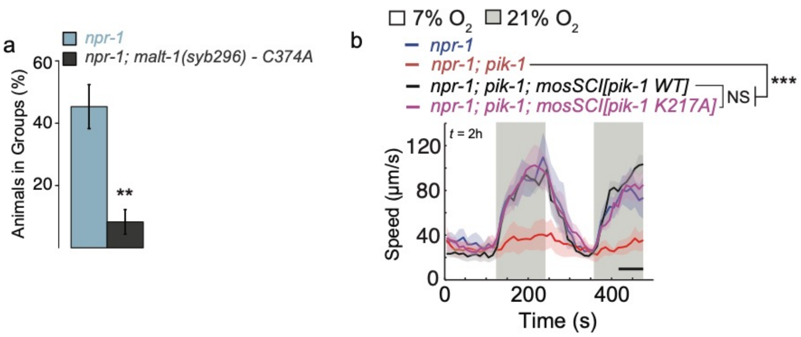

Supplementary Figure 6. Related to Figure 5. Targeted disruption of the protease activity of MALT-1 and kinase activity of PIK-1/IRAK.

a *malt-1(syb296)* mutants expressing catalytically inactive MALT-1 C374A exhibit strong aggregation defects compared to *npr-1* animals. N = 5 assays, **, P = 0.0018, one-way ANOVA with Tukey's post hoc HSD.

b PIK-1 ATP-binding may not be required for avoidance of 21% O_2. Single copy transgenes (MosSCI) expressing *pik-1* WT and *pik-1 K217A* cDNA rescue the O_2-response defect of *pik-1(tm2167)* mutants equally well. K217 corresponds to the lysine residue coordinating ATP binding in kinase active sites. n = 40 animals (*npr-1*), n = 69 animals (*npr-1; pik-1*), n = 70 animals (*npr-1; pik-1; mosSCI[pik-1 K217A]*), n = 56 animals (*npr-1; pik-1; mosSCI[pik-1 WT]*). Plots show average speed (line) and SEM (shaded regions). NS, P = 0.09, ***, P < 0.001, two-sided Mann-Whitney U test.

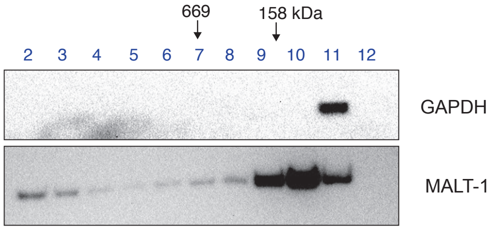

Supplementary Figure 7. Related to Figure 6**.** The elution profile MALT-1 and GAPDH proteins in *C. elegans* extract run on a Superose 6 Gel Filtration column and visualized by immunoblot. Unlike GAPDH control, MALT-1 is found in high-molecular weight fractions. This experiment was performed once.

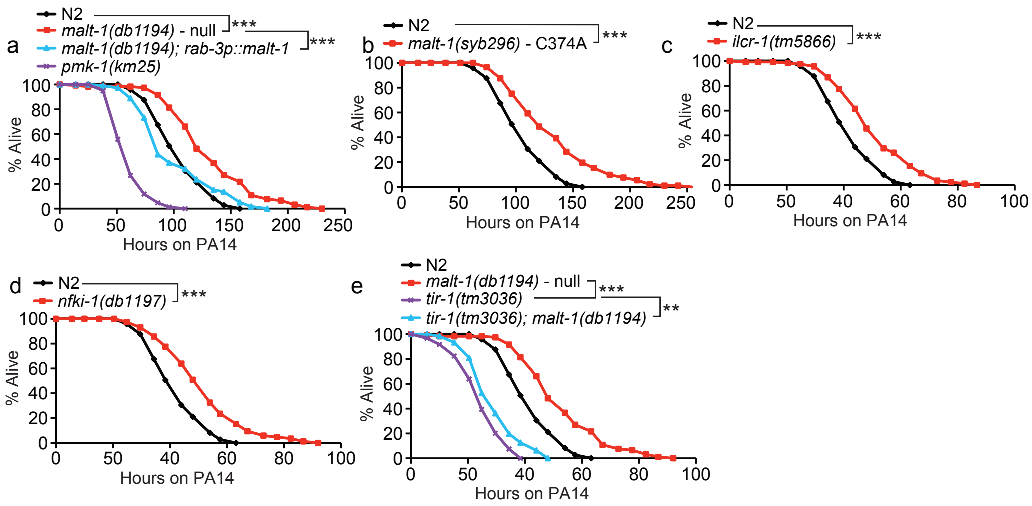

Supplementary Figure 8. Related to Figure 8. *P. aeruginosa* PA14 small lawn assays.

a-d Mutants defective in *malt-1* and other IL-17 signaling components are resistant to *P. aeruginosa* PA14. The enhanced survival in PA14 small lawn assays of *malt-1* mutants compared to N2 controls is rescued by pan-neuronal expression of *malt-1* gDNA. n ≥ 74 animals. *, P, < 0.05, **, P, < 0.01, ***, P, < 0.001, logrank test. Precise n numbers and P values are provided in Supplementary Table 2.

e The enhanced resistance of *malt-1* mutants to PA14 requires TIR-1. Like *tir-1* mutants, *malt-1; tir-1* double mutants are hypersensitive to PA14 infection. n ≥ 95 animals. ***, P < 0.001, logrank test. Precise n numbers and P values are provided in Supplementary Table 2.

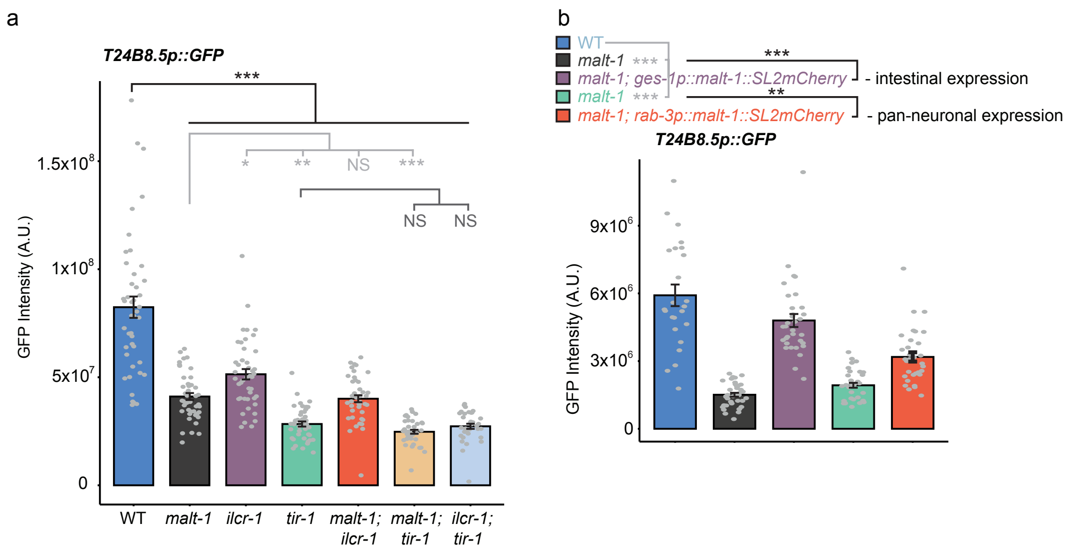

Supplementary Figure 9. Related to Figure 8. MALT-1 regulates innate immune response gene expression.

a Expression from *T24B8.5p::GFP*, a reporter of the innate immune response, is inhibited in *malt-1(db1194)*, *ilcr-1(tm5866)*, and *tir-1(tm3036)* mutants compared to N2. n = 44 animals (WT, *malt-1*, and *ilcr-1*), n = 42 animals (*tir-1*), n = 43 animals (*malt-1*; *ilcr-1*), n = 39 animals (*malt-1*; *tir-1*), n = 35 animals (*ilcr-1*; *tir-1*). *, $P < 0.05$, **, $P < 0.01$, ***, $P < 0.001$, one-way ANOVA with Tukey's post hoc HSD.

b *T24B8.5p::GFP* expression in *malt-1* mutants is restored by tissue-specific expression of WT *malt-1* cDNA in the intestine (*ges-1* promoter) or the nervous system (*rab-3* promoter). n = 42 animals (blue bars), n = 37 animals (black bars), n = 33 animals (purple bars), n = 24 animals (green and red bars). **, $P < 0.01$, ***, $P < 0.001$, one-way ANOVA with Tukey's post hoc HSD.

Fig. 6a

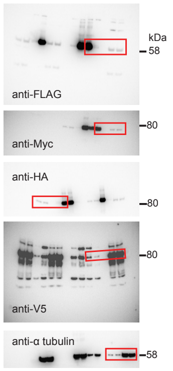

Fig. 6d

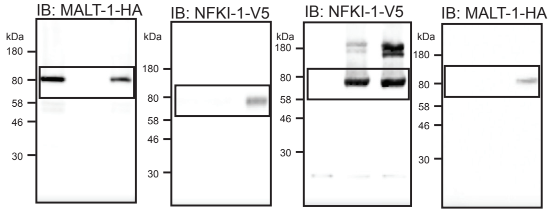

Fig. 6e

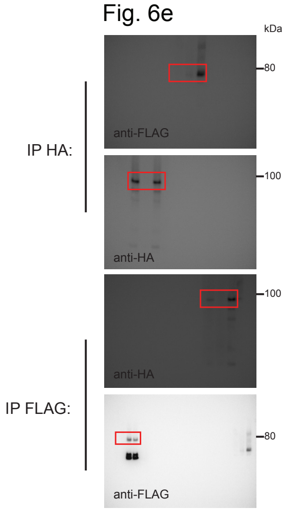

Fig. 6g

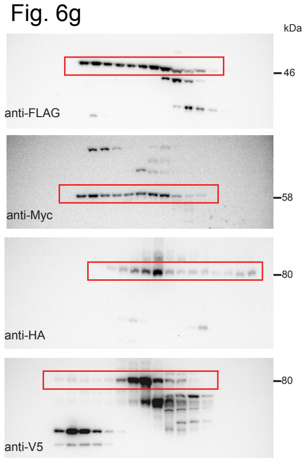

Fig. 7a

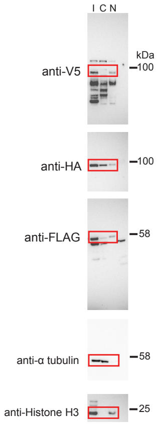

Supplementary Fig. 7

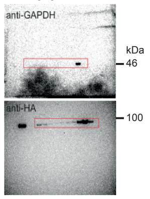

Supplementary Figure 10. Uncropped Western blots (original scans).

Supplementary Tables

Supplementary Table 1 Related to Fig. 8**.** Summary of PA14 big lawn Survival data analysis of IL-17 pathway mutants.

**Strain**	**Mean lifespan ±s.e.m. (hours)**	**Age in hours at 50% mortality**	**n (died/total)**	**Bonferroni *p* value vs. N2**
N2	48.47 ± 0.8	48	83/120	
malt-1(db1194)	56.95 ± 1.35	54	91/120	6.9e-7
malt-1 (db1194); rab-3p:: malt-1	45.25 ± 0.86	48	86/120	0.069 (0*)
pmk-1 (km25)	36.91 ± 0.62	38	104/120	0
malt-1(syb296)	62.16 ± 1.36	62	81/120	0
ilcr-1 (tm5866)	57.82 ± 1.25	54	87/120	0
nfki-1 (db1197)	57.72 ± 1.31	54	85/120	7.7e-8
tir-1(tm3036)	41.1 ± 0.81	38	98/120	1.6e-7 (0*)

malt-1(db1194); tir-1(tm3036	45.2 ± 1.48	38	98/120	0.0319 (0.0000014*) (0.2221**)

*p value against *malt-1(db1194)*. ** p value against *tir-1(tm3036)*.

Supplementary Table 2 Related to Fig. 8**.** Summary of PA14 small-lawn survival data analysis of IL-17 pathway mutants.

Strain	Mean lifespan ±s.e.m. (hours)	Age in hours at 50% mortality	n (died/total)	Bonferroni *p* value vs. N2
N2	105.18 ± 2.31	110	95/120	
malt-1(db1194)	132.14 ± 3.67	120	96/120	0
malt-1 (db1194); rab-3p:: malt-1	101.31 ± 3.74	86	74/120	1 (1e-7*)
pmk-1(km25)	61.73 ± 1.34	62	112/120	0
malt-1(syb296)	132.92 ± 3.92	120	98/120	2.9e-8
ilcr-1(tm5866)	127.20 ± 3.46	120	91/120	6.8e-7
nfki-1(db1197)	129.17 ± 3.54	135	94/120	2.1e-7
tir-1(tm3036)	73.82 ± 2.1	74	100/120	0 (0*)
malt-1(db1194); tir-	62.19 ± 1.88	62	99/120	0

1(tm3036				(0*)<br><br>(0.0039**)

*p value against *malt-1(db1194)*.   ** p value against *tir-1(tm3036)*.

Supplementary Table 3 Related to Fig. 8. Summary of lifespan of IL-17 pathway mutants on OP50.

Strain	Mean lifespan ±s.e.m. (days)	Age in hours at 50% mortality	n (died/ total)	Bonferroni *p* value vs. N2	Independent replicate 1 (n; mean lifespan ±s.e.m. (days); Bonferroni *p value*)	Independent replicate 2 (n; mean lifespan ±s.e.m. (days); Bonferroni *p value*)	Independent replicate 3 (n; mean lifespan ±s.e.m. (days); Bonferroni *p value*)
N2	18.68 ± 0.31 (Figure 6i-l)	19	157/ 180		134/180; 17.32 ± 0.37	142/180; 18.7 ± 0.31; (Figure 6m)	143/180; 20.25 ± 0.54
malt-1 (db1194)	22.76 ± 0.41 (Figure 6j)	23	132/ 180	0	112/180; 20.84 ± 0.58; 2.9e-7	146/180; 21.29 ± 0.3; 1.3e-7 (Figure 6m)	155/180; 25.62 ± 0.63; 0
malt-1 (db1194); rab-3p:: malt-1	18.56 ± 0.38 (Figure 6j)	19	109/ 180	1 (0*)	146/180; 18.78 ± 0.46; 0.0105 (0.0115*)		
ilc-17.1 (tm5218)	20.72 ± 0.35 (Figure 6k)	21	140/ 180	0.0001	117/180; 19.00 ± 0.45; 0.0097		112/180; 24.06 ± 0.83;; 0.0001
illc-17.1 (tm5218); ilc-17.1p::	13.78 ± 0.25 (Figure 6k)	13	116/ 180	0 (0*)	104/180; 13.84 ± 0.31; 0 (0**)	96/180; 16.11 ± 0.32; 0.000006	105/180; 16.56 ± 0.5; 0.000004

ilc-17.1						(Figure 6m)	(0**)
ilc-17.1 (tm5218); malt-1 (db1194)	21.62 ± 0.41 (Figure 6l)	23	111/ 150	1.2e-7 (0.2044*) (0.3839**)			131/180; 23.95 ± 0.64; 0.0001 (0.1232*) (1**)
malt-1(db1194); ilc-17.1p:: ilc-17.1						92/180; 21.48 ± 0.34; 4.5e-8 (1*) (0***) (Figure 6m)	116/180; 23.95 ± 0.71; 0.0002 (0.1847*) (0***)

**p* value against *malt-1(db1194)*. ***p* value against *ilc-17.1(tm5218)*.

****p* value against *ilc-17.1(tm5218)*; *ilc-17.1p:: ilc-17.1*

Supplementary Table 4 Knockin alleles used in this study.

>malt-1(syb296)

C374A

Synonymous mutation

ttaccccactagcactactcctccttcaataattttttatttcagAAAATGAACTACAAAACAGTAACTCTCGCTGATCTGACACTT
GATGAAATGAGATATTTTATTAGAGTTTATCAGAAATTGATTGGAAATGGAGTATACGgtaggtatatttgatttttaa
aaatataaaaaagaacacttaatttagCTGTATTCTATTTTGTTGGTCATGGATTTGAAGTGAATGGTCAATGTTATTT
ACTTGGTGTTGATGCTCCAGCAGATGCTCATCAACCACAACATTCAATGAGTATGGATTGGgtaaatacctat
aaaaactatatttagagaatctaaatccaattccagCTTTTGTCAATTTTCCGTCATAAAACTCCTGATTTAAACTTGCTT
CTTCTTGATGTCGCGCGCAAATTTGTTCCgtaagtattaaaactgaattcgcgtaactctgtcaaattcgttaaatttaaaataattcg
aaaagatattatgatcagaacatttttatttgaaaaccggcaacaaatttctcaaattccacatttgccaaaaatttggcagtatgccaaaactttagtatacg
gtatttagaaaaatcgccagcttgaacagttgaatatttccagATATGATGCGATCAGTGCATTTGTGGAATATTCAGAACAAT
TCAAAAAAATTCCATCGAGCCCATAGAAATATGGTTTATGGATACTCTACAAGCGGTGGAGTCGGAGCAT
ATGAGgtttctttttcatttgaaaaaagtttataaaaattgattattttcagGTTAAAGGAGAAGTGAATGGTGTCTTTATGAAATAT
TTGAAGAATCATGTTCAATTGGAAATATCTGTTATTGATATGTTGAATAAAGTACTATTAGgtaagtatatgaactt
tttctctgagaactatacactatccaaagtttctactctgatttatatttttgcagATATTGGGGATGATCAAAAAGTTTGTGATCTTCAA
GTTCCCGAG

>malt-1(syb573)

3XHA + Twin-Strep

Synonymous mutation

CTCAACAATTAGTCGCTACGATTTGGTTTCAATTTTGTGGAAATTTCACAAACAAGgtttctagaaaatgtcctcaatt
ctttaaagtataaaatattcagGTTTACGTATTCGCATCAATATCAGACTTCCGTCCTTGCCAGGAAGACACCGATA
TGGGAGAAAATGAAGAATTAAGTGAAAATGCATTGAATCATCGAGCATTTGTTGAATTTCCTGAAGAGCT
TCATTGTTCTGATGTTCGAGAATATAATGATGATGAAGAAGGTGTCTCAATGTTATGGATTCTATCAGGA
TTACAAAAAAATCAAGAAAGAAGCCGGATTGACATGTGAAGTTCATTTAAGACACGTTGATGATCCAGAA
AAGACGATTGAGATGAAGAATGTTGACATTGGTCATATTTTGATTACAAGAATCAAATGTCTACAGTATC
CATACGACGTCCCAGATTATGCTTACCCGTACGACGTTCCAGACTACGCATATCCATATGATGTCCCAG
ACTACGCTTGGTCGCACCCGCAATTTGAGAAAGGATCCGCCGGATCCGCCGCCGGATCCGGAGCCGG
ATGGTCCCATCCACAGTTCGAAAAGTAAtgcattgccatgtgaacacctacgggtccgaaaatccattattaattatttttgccaattgaa
atgtagtactaatgaaacgaaatctcatttattttacaatgaataaaatgcgtaaagtaatggtgtatgaattaatataaaacgaataaaccgatggaagg

agaaaaaaatgaacgaatacgccatcgtgaatgaatgaaacagacaaagcaatgggaaagaaacgacaaaaaggatgagaaagagattttcgc
gtcaatatatagagaaaatgattggacggtgtggaga

>pik-1(syb378)

linker-2XMyc

Synonymous mutation

AAAAGCATCCATTAATTGCTTCACATATCAAGGGAACACTTGCTTATCTTGCACCAGAATTCATTACATC
AAAGATTCTTACCACAAAACTTGATGTCTATAGTTTTGGAATAGTACTTTTGGAAATTGCATCTGGTCAAC
GGGCATATTCGGATTCTCGTGAAACTCGGGGGCTCGTTGAATATTGTCAGGTTAATAAGGAATTGGCAG
CACATCGGAAGATTCCAGTCAGAGAGATTTTTATTGATCGACGAGCGCCGCCACTTGTTGGTGATGAG
GAAAAATCATTTTTGGATGCTCTGATTGAAGTTGGATTAGCTGGAGCGAATAACGATCGGAAAGTTCGA
CCGACTATGTCACAAATTGTTGAATATCTTTGTAAAAATTCAATTCCGCCAGTTGTCGGAGGCGGT
GAACAAAAGTTGATATCCGAAGAGGACCTTGAGCAGAAGCTGATAAGTGAA
GAAGACCTGTAAttaatactgtgtaccttgccatattcctcgaactcgaaattttgccatttttgagtcaaaactacggtagcgggtctcgacac
gaccgcctatagagattactgtagcaggggctttttggcaaatttttatatccggcaatttgcctatttgccggaaatttcaattctggcaatttgccaatttgcct
gaaagtttcatttccggcaattttccgatttgccgaaagttttcatatccggcaatatgctgatttgccggaaatttttattttctgaaa

>actl-1(syb412)

3XFLAG

Synonymous mutation

tagagttttctcattacgaacgtcggaattttgtgatcatttttggtctgataaaataaagactcaaatatagctgctccaccttctacaaaatttttttgattaatttgt
cttaacatcacatttaaaaacttcgtttttaattaaagattgcagtatctttctgaaaatacatggctggacatctaactctctggcttccaaaaatgtttacactg
tgtgaaatgtgtattatagGAAGTGGAAAAAGTAACGGACACGTACTGGGCAGCTGTAACGAATCAATATCCATTC
CCCGGAAGTTTTGAACCATTTGTGAAGCGTCTTCTGAGAGATGGAAAAGTGAAGAAACAATCACACAAT
AATGCAAATGATCATCACGAGGATTCCATGAACTACAGTATTACACAAGGTGGTGGTGATTACA
AGGATCACGACGGAGACTATAAAGATCATGATATCGACTACAAGGATGATG
ATGATAAGTAGacaccatgtggtattttgagccaataatctatacattgtagcatctgatgaacttttttttcattgtacactttttgatgacagtcac
gtgtttttttagatttctcagacagtctttactatccacatttttcatattacaaaaacagtatttttctttcaattttctcagttaatctattaagatgttgtttccgactttc
aatttccagattttattatacactaatgaaataaatcttacagaaggttc

>*nkfi-1(syb617)*

V5

Synonymous mutation

TGATGGATGAGCAGACACAGCACAAAATTGACAACAAGTCCATCATCGAAATGCTCATCAAGGCTGGAG
CAGATCCAACGATTGCTgtaagtggattatgtgcttttgatgattaccaatacagctcttttcagGAAACTTCAACTGGAAAGACAA
TTGTACATCATGCTGTCGACAAGATGGACGTGGAACTTTTAGATgtaagttccgaatttcctcgagtttccttaataaataaat
atttaaaattttcagTTCTTAAAAACCGTTGTCAACGAAGACACGTTCACCGAGTTGGCAAATCTCTCCGACTTC
CATGGAGACACCGCAGTCGACCTTTTGTGTAGTTCCACCCAGAACGAGGACACGAATAATGTCCGCGA
AAATCTATACATCCGACTCCTGACC**AGT**GGTGCAGTCCCGAACAAGTCGAGAGCTGGAAAGCCAA
TCCCAAACCCACTTCTTGGACTCGACTCCACCTGAgctctattcagcttcattcacattttttttgacgttgttg
cataatgccatcaaaaccacctatatttgactcttcgttatagtttattttttattttgttgatctcaaaaccacccctctaatctaaaaaattaaactttgaaaataa
tcaaaaaattattttttaaaagtacttcattaaaattaatgtgttcatttttccttacaacgaccgttaagctattccactcaacgattgttaagtctacagttcaac
ctctcaattgaatattgaaaataattatttaaaacgtttacacctccaacttcaacg

Supplementary Table 5 Strain list.

Strain	Genotype
N2	Wild-type Bristol strain
AX204	*npr-1(ad609)* X
PHX412	*actl-1(syb-412) npr-1(ad609)* X
AX7213	*pik-1(syb-378)* II; *npr-1(ad609)* X
AX6130	*npr-1(ad609) nfki-1(db1198)* X*; dbIs38[nfki-1p::nfki-1::GFP, ccRFP]*
AX6145	*npr-1(ad609)* X*; dbIs38[nfki-1p::GFP, ccRFP]*
AX6969	*malt-1(db1194)* II; *npr-1(ad609)* X; *dbIs16[rab-3p::malt-1(gDNA)::GFP, ccRFP]*
AX288	[*lon-2*(*e678*) *npr-1*(*ad609*)]
AX5877	*malt-1(db1194)* II; *npr-1(ad609)* X
AX6742	*malt-1(db1194)* II; *npr-1(ad609)* X; *dbEx983[rab-3p::malt-1(cDNA)::SL2mCherry, ccGFP]*
AX6392	*malt-1(db1194)* II; *npr-1(ad609)* X; *dbEx925[rab-3p::malt-1(gDNA)::GFP, ccRFP]*
AX7502	*dbEx1097[malt-1p(4kb)::malt-1(gDNA)::GFP, ccRFP]*
AX7591	*ynIs49[flp-5::GFP]; dbEx1119[malt-1p::(4kb)::malt-1(gDNA)::GFP, rol-6(su1006)]*
AX6740	*malt-1(db1194)* II; *npr-1(ad609)* X*; dbEx982[gcy-32p::malt-1(cDNA)::SL2mKate, ccGFP]*
AX7109	*malt-1(db1194)* II; *npr-1(ad609)* X*; dbEx1054[flp-5p::malt-1(cDNA):SL2mKate, ccRFP]*

AX7132	*malt-1(db1194)* II; *npr-1(ad609)* X*; dbEx982[gcy-32p::malt-1(cDNA)::SL2mKate, ccGFP]; dbEx1054[flp-5p::malt-1(cDNA):SL2mKate, ccRFP]*
AX5995	*malt-1(db1194)* II; *npr-1(ad609)* X*; dbEx637*[*RMGp::YC2.60, ccRFP*]
AX6836	*malt-1(db1194)* II; *npr-1(ad609)* X; *dbEx637*[*RMGp::YC2.60, ccRFP*]; *dbEx996[npr-1p::malt-1::SL2mCherry, ccRFP]*
AX5989	*malt-1(db1194)* II; *npr-1(ad609)* X*; dbEx614*[*gcy-37p::YC2.60, ccRFP]*
AX5797	*npr-1(ad609) ilc-17.1(tm5218)* X*; dbEx614*[*gcy-37p::YC2.60, ccRFP]*
AX5811	*npr-1(ad609) ilc-17.1(tm5218)* X*; dbEx614*[*RMGp::YC2.60, ccRFP]*
AX6984	*malt-1(db1194)* II*; npr-1(ad609) ilc-17.1(tm5218)* X; *dbEx614*[*RMGp::YC2.60, ccRFP]*
AX5689	*npr-1(ad609) ilc-17.1(tm5218)* X
AX6727	*malt-1(db1194)* II; *npr-1(ad609)* X; *dbEx978[hsp-16.41p::malt-1 ::SL2mCherry, ccRFP]*
AX7149	*malt-1(syb296)* II; *npr-1(ad609)* X
AX7221	*malt-1(syb296)* II; *npr-1(ad609)* X; *dbEx1070[rab-3p::malt-1(cDNA)::SL2mKate, ccRFP]*
AX6415	*malt-1(db1194)* II; *npr-1(ad609)* X; *dbEx930[rab-3p::malt-1(C374A)::SL2mCherry, ccRFP]*

AX6991	*malt-1(db1194)* II; *npr-1(ad609)* X; *dbEx1026[rab-3p::malt-1(C374A gDNA)::GFP, ccRFP]*
AX7006	*malt-1(db1194)* II; *npr-1(ad609)* X; *dbIs18[rab-3p::malt-1(C374A gDNA)::GFP, ccRFP]*
AX7462	*malt-1(syb573)* II; *pik-1(syb378)* IV; *npr-1(ad609) actl-1(syb412) nfki-1(syb617)* X
AX7464	*malt-1(syb573)* II; *pik-1(syb378)* IV; *npr-1(ad609) actl-1(syb412) ilc-17.1(tm5218) nfki-1(syb617)* X
AX7229	*pik-1(syb378)* IV; *npr-1(ad609) actl-1(syb412)*
PHX617	*nfki-1(syb617)* X
AX6250	*malt-1(db1194)* II; *npr-1(ad609) nfki-1(db1198)* X; *dbIs38[nfki-1p::nfki-1::GFP, ccRFP]*
AX6142	*pik-1(tm2167)* IV; *npr-1(ad609) nfki-1(db1198)* X; *dbIs38[nfki-1p::nfki-1::GFP, ccRFP]*
AX7849	*npr-1(ad609) ilc-17.1(tm5218)* X; *dbIs16[rab-3p::malt-1(gDNA)::GFP, ccRFP]*
AX5663	*ilcr-1(tm5866)* IV*; npr-1(ad609)* X
AX7850	*ilcr-1(tm5866)* IV*; npr-1(ad609)* X; *dbIs16[rab-3p::malt-1(gDNA)::GFP, ccRFP]*
AX6008	*npr-1(ad609) actl-1(db1203)* X
AX7040	*npr-1(ad609) actl-1(db1203)* X; *dbIs16[rab-3p::malt-1(gDNA)::GFP, ccRFP]*
AX5909	*pik-1(tm2167)* IV*; npr-1(ad609)* X

AX7637	*pik-1(tm2167)* IV; *npr-1(ad609)* X; *dbIs16[rab-3p::malt-1(gDNA)::GFP, ccRFP]*
AX7235	*npr-1(ad609) nfki-1(db1197)* X; *dbIs16[rab-3p::malt-1(gDNA)::GFP, ccRFP]*
AX6010	*npr-1(ad609) nfki-1(db1197)* X
AX7586	*ilc-17.1(tm5218)* X
AX7450	*ilc-17.1(tm5218)* X; *dbIs39[ilc-17.1p::ilc-17.1::mCherry]*
AX7585	*malt-1(db1194)* II
AX7449	*malt-1(db1194)* II; *dbIs16[rab-3p::malt-1(gDNA)::GFP, ccRFP]*
KU25	*pmk-1(km25)* IV
PHX296	*malt-1(syb296)* II
AX5949	*ilcr-1(tm5866)* IV
AX7587	*nfki-1(db1197)* X
TM3036	*tir-1(tm3036)* III
AX7649	*malt-1(db1194)* II*; tir-1(tm3036)* III
AX7847	*malt-1(db1194)* II*; dbIs39[ilc-17.1p::ilc-17.1::mCherry]*
AX7848	*malt-1(db1194)* II*; ilc-17.1(tm5218)*
AX6859	*malt-1(db1194)* II; *npr-1(ad609)* X*; dbEx996[npr-1p::malt-1(cDNA)::SL2mCherry, ccRFP]*
AX6134	*malt-1(db1194)* II; *pik-1(tm2167)* IV; *npr-1(ad609)* X*; dbEx637*[*RMGp::YC2.60, ccRFP*]
AX7185	*ttTi5605 II; unc-119(ed3) III; pik-1(tm2167)* IV; *npr-1(ad609)* X; *MosSCI[unc-119(+); pik-1p::pik-1(K214A)::unc-54 3'UTR]*

AX6666	*ttTi5605 II; unc-119(ed3) III; npr-1(ad609); pik-1(tm2167); MosSCI[unc-119(+); pik-1p::pik-1::unc-54 3'UTR]*
AX6829	malt-1(db1195); npr-1(ad609); *iaIs25[gcy-37p::GFP(+) unc-119(+)] dbEx979[malt-1p(4kb)::malt-1(gDNA)::mCherry, ccGFP]*
AX7479	*agIs219[T24B8.5p::GFP::unc-54-3' UTR + ttx-3p::GFP::unc-54-3' UTR]* III
AX7480	*malt-1(db1194)* II*; agIs219[T24B8.5p::GFP::unc-54-3' UTR + ttx-3p::GFP::unc-54-3' UTR]* III
AX7852	*ilcr-1(tm5866)* IV; *agIs219[T24B8.5p::GFP::unc-54-3' UTR + ttx-3p::GFP::unc-54-3' UTR]* III
AX7595	*tir-1(tm3036)* III; *agIs219[T24B8.5p::GFP::unc-54-3' UTR + ttx-3p::GFP::unc-54-3' UTR]* III
AX7871	*malt-1(db1194)* II*; ilcr-1(tm5866)* IV*; agIs219[T24B8.5p::GFP::unc-54-3' UTR + ttx-3p::GFP::unc-54-3' UTR]* III
AX7872	*malt-1(db1194)* II*; tir-1(tm3036)* III*; agIs219[T24B8.5p::GFP::unc-54-3' UTR + ttx-3p::GFP::unc-54-3' UTR]* III
AX7854	*tir-1(tm3036)* III*; ilcr-1(tm5866)* IV*; agIs219[T24B8.5p::GFP::unc-54-3' UTR + ttx-3p::GFP::unc-54-3' UTR]* III
AX7572	*malt-1(db1194)* II*; agIs219[T24B8.5p::GFP::unc-54-3' UTR + ttx-3p::GFP::unc-54-3' UTR]* III; *dbEx110[rab-3p::malt-1::SL2mCherry, ccRFP]*

AX7573 *malt-1(db1194)* II*; agIs219[T24B8.5p::GFP::unc-54-3' UTR + ttx-3p::GFP::unc-54-3' UTR]* III; *dbEx111[ges-1p::malt-1::SL2mCherry, ccRFP]*

Supplementary Table 6 Primers used in this study.

Primers for cloning *malt-1* promoter, cDNA and gDNA:

malt-1 promoter F	ggggACAACTTTGTATAGAAAAGTTGctgccggtggattccaacatattg
malt-1 promoter R	ggggACTGCTTTTTTGTACAAACTTGtctgaaattggggttcaagaaatttatttttgatttttaaaata
malt-1 ORF F	ggggACAAGTTTGTACAAAAAAGCAGGCTtttcagaaaaatgaacacaaacttggcggagtt
malt-1 ORF R	ggggACCACTTTGTACAAGAAAGCTGGGTATTACTGTAGACAT
malt-1(C374A) F	TCTTGATGTCgcCAGAAAATTTGTTCCATATG
malt-1(C374A) R	gcgcgtcaagttgtGCCTGACGACGAGTTGTGCTGTTTTAGAGCTAGAA

Oligos for generating sgRNA expression plasmid used to make *db1194*:

malt-1 sgRNA EcoRI 1	gcgcgtcaagttgtGgatcaggtatccaccgtagGTTTTAGAGCTAGAA
malt-1 sgRNA EcoRI 2	TTCTAGCTCTAAAACctacggtggatacctgatcCacaacttgacgcgc

Primers for cloning with *E. coli* expression plasmids:

MALT-1-HA F	ATATATGCTCTTCTAGTAACACAAACTTGGCGGAGTTACCTG
MALT-1-HA R	TATATAGCTCTTCATGCAGCATAATCTGGTACGTCGTATGGGTATCCTCCTC CCTGTAGACATTTGATTCTTGTAATCAAAATATGACC
ACTL-1-FLAG F	ATATATGCTCTTCTAGTACTAAGATGAAAATGGACGTAACAATTGAGTTGG
ACTL-1-FLAG R	TATATAGCTCTTCATGCCTTGTCATCGTCGTCCTTGTAATCTCCTCCTC-CTTGTGTAATACTGTAGTTCATGGAATCCTCG
NFKI-1-V5 F	ATATATGCTCTTCTAGTGCAACCGTTGCCCCCAAGGGAAACTGC
NFKI-1-V5 R	TATATAGCTCTTCATGCCGTAGAATCGAGACCGAGGAGAGGGTTAG-GGATAGGCTTACCTCCTCCTCCAGCTCTCGACTTGTTCGGGACTGC

Primers for cloning with *S. cerevisiae* Y2H plasmids:

NFKI-1(full) EcoRI/BamHI 1	GCGAATTCATGGCAACCGTTGCCCC
NFKI-1(full) EcoRI/BamHI 2	GCGGATCCTCAAGCTCTCGACTTGTTCGG
NFKI-1(1-374) EcoRI/BamHI 1	GCGAATTCATGGCAACCGTTGCCCC
NFKI-1(1-374) EcoRI/BamHI 2	GCGGATCCTCATGCATCGCGGTTAGTAAG
MALT-1(full) XmaI/XhoI 1	GTTCCCGGGGATGAACACAAACTTGGCGG
MALT-1(full) XmaI/XhoI 2	CGGCTCGAGTTACTGTAGACATTTGATTCTTGT
MALT-1(1-81) XmaI/XhoI 1	GTTCCCGGGgATGAACACAAACTTGGCGG
MALT-1(1-81) XmaI/XhoI 2	CGGCTCGAGTTATCTTGAAAGAAACTGCAATCT
MALT-1(248-639) XmaI/XhoI 1	GTTCCCGGGgCGAGCAGCAGATAAAGTTG
MALT-1(248-639) XmaI/XhoI 1	CGGCTCGAGTTACTGTAGACATTTGATTCTTGT
