# Supplementary material for: MALT-1 mediates IL-17 neural signaling to regulate C. elegans behavior, immunity and longevity
Source: Nat Commun. 2020 Apr 29;11:2099. doi: 10.1038/s41467-020-15872-y (PMC7190641; doi:10.1038/s41467-020-15872-y)
Supplement: Supplementary file 3 — Description of Additional Supplementary Files [file 41467_2020_15872_MOESM3_ESM.pdf]

Description of Additional Supplementary Files

Supplementary Data 1. Related to Figure 1. LC-MS/MS analysis of proteins co-immunoprecipitating with IL-17 signaling components. Shown are total spectral counts for all proteins that specifically co-IP with ACTL-1 (**a**), PIK-1 (**b**), NFKI-1 (**c**), and MALT-1 (**d**).

Supplementary Data 2. Related to Figure 6. Peptides derived from MALT-1 and PIK-1 detected in quantitative LC-MS/MS of proteins that interact with NFKI-1::GFP (Fig. 6b and c).

Supplementary Data 3. Related to Figure 8. Whole-animal gene-expression profiles of *ilc-17.1(tm5218)*; *npr-1, malt-1(db1194)*; *npr-1,* and *nfki-1(db1197); npr-1* mutants compared to *npr-1* controls.

a and **b,** All genes significantly downregulated (**a**) and upregulated (**b**) in all three conditions compared to control. FPKM values, fold-change and statistics for each gene are shown for the *npr-1* vs *npr-1; ilc-17.1(tm5218)* comparison.

c and **d,** Significantly enriched GO (**c**) and KEGG (**d**) terms for genes whose expression is significantly altered in all three mutants compared to control, with a $\log_2$ fold-change cut-off of 0.25.

Supplementary Data 4. Related to Figure 8. Comparison of neuropeptide gene expression in mutants defective in response to 21% O_2[30], and mutants defective in IL-17 signaling.

Supplementary Data 5. Related to Figure 8. Statistical analysis of survival data for IL-17 pathway mutants on PA14. Shown are Kaplan-Meier analysis and logrank tests for big lawn (**a** and **b**) and small lawn (**c** and **d**) assays.

Supplementary Data 6 Related to Figure 8. Statistical analysis of survival data for IL-17 pathway mutants on OP50. Shown are Kaplan-Meier analyses (**a**-**d**) and logrank tests (**e**-**h**).
