# Supplementary material for: MALT-1 mediates IL-17 neural signaling to regulate C. elegans behavior, immunity and longevity
Source: Nat Commun. 2020 Apr 29;11:2099. doi: 10.1038/s41467-020-15872-y (PMC7190641; doi:10.1038/s41467-020-15872-y)
Supplement: Supplementary file 10 — Reporting Summary [file 41467_2020_15872_MOESM10_ESM.pdf]

nature research

Corresponding author(s): Mario de Bono

Last updated by author(s): Mar 3, 2020

Reporting Summary

Nature Research wishes to improve the reproducibility of the work that we publish. This form provides structure for consistency and transparency in reporting. For further information on Nature Research policies, see Authors & Referees and the Editorial Policy Checklist.

Statistics

For all statistical analyses, confirm that the following items are present in the figure legend, table legend, main text, or Methods section.

n/a	Confirmed	
☐	☒	The exact sample size (*n*) for each experimental group/condition, given as a discrete number and unit of measurement
☐	☒	A statement on whether measurements were taken from distinct samples or whether the same sample was measured repeatedly
☐	☒	The statistical test(s) used AND whether they are one- or two-sided<br>*Only common tests should be described solely by name; describe more complex techniques in the Methods section.*
☒	☐	A description of all covariates tested
☐	☒	A description of any assumptions or corrections, such as tests of normality and adjustment for multiple comparisons
☐	☒	A full description of the statistical parameters including central tendency (e.g. means) or other basic estimates (e.g. regression coefficient) AND variation (e.g. standard deviation) or associated estimates of uncertainty (e.g. confidence intervals)
☐	☒	For null hypothesis testing, the test statistic (e.g. *F*, *t*, *r*) with confidence intervals, effect sizes, degrees of freedom and *P* value noted<br>*Give P values as exact values whenever suitable.*
☒	☐	For Bayesian analysis, information on the choice of priors and Markov chain Monte Carlo settings
☒	☐	For hierarchical and complex designs, identification of the appropriate level for tests and full reporting of outcomes
☒	☐	Estimates of effect sizes (e.g. Cohen's *d*, Pearson's *r*), indicating how they were calculated

Our web collection on statistics for biologists contains articles on many of the points above.

Software and code

Policy information about availability of computer code

Data collection	Locomotory responses were recored with FlyCapture 1.X (FLIR Systems). NIS-elements (Nikon) was used to record FRET levels (YC2.60), and capture images of animals expressing T24B8.5p::GFP. Images showing MALT-1 expression were captured using LAS X (Leica). Unicorn 7.0 (GE Healthcare Life Sciences) was used for size-exclusion chromatography. Western blots were imaged with Image Lab 4.1 (Bio-Rad).
Data analysis	Locomotory responses were quantified using Zentracker (https://github.com/wormtracker/zentracker), a custom Matlab software. FRET levels (YC2.60) were quantified using Neuron Analyzer (https://github.com/neuronanalyser/neuronanalyser), a custom Matlab software. ANOVA tests were performed using common functions in RStudio (v 1.0.143). FIJI (ImageJ v2.0.0-rc-69) was used to obtain of Z-stacks of MALT-1 expression patterns, and quantify T24B8.5p::GFP expression. Kaplan-Meier analyses and logrank tests were performed using OASIS (https://sbi.postech.ac.kr/oasis/). Mascot v2.4.0 (Matrix Science), and Scaffold v4.10.0 (Proteome Software Inc) were used to analyse LC-MS/MS data. TMT-labeled samples were processed using MaxQuant with the integrated Andromeda search engine (v.1.5.5.1), and analyzed using Perseus (v 1.5.5.0). CloudMap (http://hobertlab.org/cloudmap/) was used to analyze WGS data, and TopHat v2.1.0 and Cufflinks v2.2.1 were used for RNA-seq analysis.

For manuscripts utilizing custom algorithms or software that are central to the research but not yet described in published literature, software must be made available to editors/reviewers. We strongly encourage code deposition in a community repository (e.g. GitHub). See the Nature Research guidelines for submitting code & software for further information.

Data

Policy information about availability of data

All manuscripts must include a data availability statement. This statement should provide the following information, where applicable:

- Accession codes, unique identifiers, or web links for publicly available datasets
- A list of figures that have associated raw data
- A description of any restrictions on data availability

Data supporting the findings of this study are available from the corresponding author.

Field-specific reporting

Please select the one below that is the best fit for your research. If you are not sure, read the appropriate sections before making your selection.

☒ Life sciences ☐ Behavioural & social sciences ☐ Ecological, evolutionary & environmental sciences

For a reference copy of the document with all sections, see nature.com/documents/nr-reporting-summary-flat.pdf

Life sciences study design

All studies must disclose on these points even when the disclosure is negative.

Sample size	Sample size and statistical test were chosen based on previous studies that used the same or similar methods;<br>For aggregation assays: de Bono, M. & Bargmann, C. I. Cell 94, 679–689 (1998).<br>For locomotory responses to O2: Laurent, P. et al. eLife 4, e04241 (2015) and Fenk, L. A. & de Bono, M. Proc. Natl. Acad. Sci. USA 112, E3525–E3534 (2015).<br>For salt chemotaxis assays: Saeki, S., Yamamoto, M. & Iino, Y. J. Exp. Biol. 204, 1757–1764 (2001).<br>For P. aeruginosa killing assays assays: Jeong, D.-E. et al. . EMBO J. 36, 1046–1065 (2017).<br>For lifespan assays: Artan, M. et al. Genes Dev. 30, 1047–1057 (2016).<br><br>No statistical method was used to select sample sizes.
Data exclusions	Data were excluded based on the following pre-established criteria:<br>-In recordings of locomotory responses worms in contact with other animals were excluded in order to ensure that only individual animals were analyzed.<br>-Recordings of FRET levels (YC2.60) were checked manually for data points in which objects other than the neuron of interest were measured; these data points were excluded in order to ensure that all data points correspond to the neuron of interest.<br>-In survival analyses, animals that died from causes other than ageing were censored according to standard methods, as in Artan, M. et al. Genes Dev. 30, 1047–1057 (2016).
Replication	All data in the manuscript was obtained with at least two biological replicates, with the exception of Fig. 1b, 1d, 6d and Supplementary Fig. 7 which were performed once. Please see the figure legends for the precise number of replicates.
Randomization	Organisms were allocated into experimental groups based on genotype, as determined by standard approaches (PCR or sequencing).<br>To control covariates, assays were run in parallel (aggregation, survival, T24B8.5p::GFP and salt chemotaxis assays), or control and experimental conditions were alternated in subsequent experimental trials (locomotory responses and Ca2+ imaging).
Blinding	Aggregation was scored blind to genotype. Locomotory responses, Ca2+ imaging and T24B8.5p::GFP were analyzed by computer code/software. In survival and salt chemotaxis assays the scorer was not blind to genotype, but phenotypes scored were strong meaning that the influence of the scorer was negligible.

Reporting for specific materials, systems and methods

We require information from authors about some types of materials, experimental systems and methods used in many studies. Here, indicate whether each material, system or method listed is relevant to your study. If you are not sure if a list item applies to your research, read the appropriate section before selecting a response.

Materials & experimental systems

n/a	Involved in the study
☐	☒ Antibodies
☒	☐ Eukaryotic cell lines
☒	☐ Palaeontology
☐	☒ Animals and other organisms
☒	☐ Human research participants
☒	☐ Clinical data

Methods

n/a	Involved in the study
☒	☐ ChIP-seq
☒	☐ Flow cytometry
☒	☐ MRI-based neuroimaging

Antibodies

Antibodies used	As described in the methods the following commercially available antibodies were used: anti-FLAG M2-Peroxidase (A8592, Sigma A8592-.2MG, diluted 1:1000), anti-Myc (9B11, Cell Signaling #2276, diluted 1:1000), anti-HA (C29F4, Cell Signaling #3724, diluted 1:1000), anti-V5 (A190, Bethyl Laboratories A190-120A, diluted 1:1000), anti-Histone H3 (Cell Signaling #9715, diluted 1:1000), anti-alpha tubulin (DM1A, abcam ab40742, diluted 1:4000), anti-Rabbit IgG (Bio-Rad #1706515, diluted 1:3000), and anti-Mouse IgG (Bio-Rad #1706516, diluted 1:3000).
Validation	Antibody validation was performed by the manufacturer. Additionally, they have been validated in the literature:

-anti-FLAG M2-peroxidase: Sato et al. Nat. Commun. 2019, 10, 5708.
-anti-Myc: Kim et al. Nat. Commun. 2019, 10, 4898.
-anti-HA: Boone et al. Nat. Commun. 2019, 10, 5490.
-anti-V5: Wang et al. Nat. Commun. 2019, 10, 3201.
-anti-alpha tubulin: Oliveira-Mateos et al. Nat. Commun. 2019, 10, 3979.
-anti-Histone H3: Enfield et al. Nat. Commun. 2019, 10, 5438.
-anti-Rabbit IgG: Roderer et al. Nat. Commun. 2019, 10, 5263.
-anti-Mouse IgG: Mariano et al. Nat. Commun. 2019, 10, 5484.

Animals and other organisms

Policy information about studies involving animals; ARRIVE guidelines recommended for reporting animal research

Laboratory animals	All C. elegans experiments were performed using hemaphrodites. Please see Supplementary Table 5 for a complete list of strains used in the is study. Young adults were used for all behavioral experiments. Mixed stage cultures were used for IP/MS experiments.
Wild animals	The study did not involve wild animals.
Field-collected samples	The study did not involve samples collected from the field.
Ethics oversight	No ethical approval was needed for this work as the invertebrate nematode C. elegans is not a sentient animal model.

Note that full information on the approval of the study protocol must also be provided in the manuscript.
